# Effects of 60 versus 30 Minutes of Pilates Exercise on Cervicothoracic Alignment, Muscle Strength, and Endurance in University Students with Upper Crossed Syndrome

**DOI:** 10.3390/jcm13154376

**Published:** 2024-07-26

**Authors:** Woo-Lim Mun, Su-Yeon Roh

**Affiliations:** Department of Exercise Rehabilitation, Gachon University, Incheon 21936, Republic of Korea; woolim@gachon.ac.kr

**Keywords:** upper crossed syndrome, Pilates, postural alignment, muscle strength, muscle endurance

## Abstract

**Background/Objectives**: This study determined the effects of 60 min or 30 min Pilates program on cervicothoracic alignment, muscular strength, and endurance in university students with upper-crossed syndrome (UCS). **Methods**: Twenty-six university students with UCS were randomly allocated into 60 min Pilates (60 PG, *n* = 9), 30 min Pilates (30 PG, *n* = 9), and control (CG, *n* = 8) groups. The 60 and 30 PG students participated in the Pilates program, focusing on relaxation, stability, mobility, and strengthening of the cervical, thoracic, and scapular muscles for 60 or 30 min per session, twice a week, for 12 weeks. Cervicothoracic alignment was measured using BodyStyle to determine craniovertebral angle (CVA), forward shoulder angle (FSA), head position angle (HPA), head tilt angle (HTA), and kyphosis angle (KA). We measured the muscular strength and endurance of the shoulder on the dominant side. **Results**: After the intervention, CVA, HPA, HTA, and KA significantly improved in the 60 and 30 PG (all *p* < 0.01) but not in the CG (*p* > 0.05). There were no significant differences between the 60 and 30 PG at 4, 8, and 12 weeks. Shoulder strength differed between shoulder flexion and horizontal abduction (*p* < 0.01). Shoulder endurance differed between extension, flexion, and horizontal abduction (*p* < 0.05, *p* < 0.01, and *p* < 0.001, respectively). **Conclusions**: This study confirmed the effectiveness of the Pilates program in improving cervicothoracic sagittal alignment and shoulder muscular function in university students with UCS. Additionally, the effects of participating in 60 and 30 min Pilates programs were found to be equivalent. Therefore, encouraging busy university students to participate in at least 30 min of the Pilates program is important.

## 1. Introduction

A sedentary lifestyle leads to decreased physical activity, which contributes to metabolic diseases such as obesity, diabetes, hyperlipidemia, and hypertension. It also contributes to musculoskeletal disorders such as muscle imbalance and pain. In terms of sedentary behavior among Koreans, both men and women showed similar rates of increased sedentary time in 2020 compared to those in 2019. In particular, in the 19–29 age group, men had an increased sedentary time from 9.0 h to 9.2 h, and women increased from 9.8 h to 10.2 h [1].

Increased sedentary time can strain muscles and cause an imbalanced posture [2]. Sitting in the wrong position for long periods can lead to muscle imbalance in the head, cervical, thoracic, and shoulder, which is known as the upper crossed syndrome (UCS) [3,4]. The upper crossed syndrome is characterized by cervical lordosis and a forward head posture (tech neck or text neck) of the cervical spine, as well as rounded shoulders and thoracic hyperkyphosis. Additionally, there is decreased stability of the glenohumeral joint and winging scapular [4]. This results in partial joint dysfunction of the atlanto-occipital joint, C4–5 segment, and T4–5 joint, with compensatory overactivation of the levator scapulae and upper trapezius muscles [5]. Imbalance and compensation of the joints and muscles lead to postural misalignment in the sagittal plane of the upper extremities [6]. Furthermore, the stability of the upper extremities is reduced, leading to decreased muscle strength and endurance around the shoulder [7]. Eventually, the posture of the UCS causes stress to the muscles and joints, resulting in a limited range of motion and pain [8].

Characterized by the muscles involved in the UCS, white muscles (deep cervical flexors, rhomboids, lower trapezius, and serratus anterior) easily lose strength under stress [4]. In contrast, the red muscles (suboccipital muscle, upper trapezius, levator scapulae, and pectoralis muscle) are overactive and hypertonic because of weakened muscles, which decrease their endurance capacity [4]. In other words, UCS is the result of an imbalanced pattern created by weak and tight muscles [4]. A study of 524 male and female university students aged 18–25 years found that prolonged computer use while sitting in an improper posture was associated with 1.64–2.18 times higher odds of experiencing neck pain [9]. Therefore, the correction of poor posture is necessary to prevent musculoskeletal disorders among university students. Furthermore, rehabilitation interventions that can improve the strength of the weakened muscles, relax tense muscles, and improve muscular endurance are required.

In general, to improve muscle imbalances caused by UCS, stretching is suggested for tight muscles to increase muscle length to normal, and strength training is suggested for weak muscles [4]. However, previous studies have shown that the effect of stretching alone on spinal alignment in adults is unclear [10]. However, a strength exercise program that includes stretching has been shown to have a positive effect on spinal posture [11]. In addition, improved core stability is known to increase whole-body strength and muscular endurance and may also have a positive impact on posture related to spinal curvature [12,13]. Weak core muscles have also been shown to result in upper extremity dysfunction [14,15]. Furthermore, shoulder pain can be easily triggered [16]. Therefore, core stability is essential to improving posture, upper extremity function, and pain due to spinal curvature alignment.

Pilates, an interventional exercise method with a proven positive effect on improving deformed posture, has been applied in various studies and sports facilities to correct client imbalances [17]. Pilates promotes core stability, which can lead to improved posture and movement [18]. A previous study found that 50 min of Pilates improved shoulder and hip postures as measured from the frontal plane in children aged 10–14 years old [19]. In adult women, 60 min sessions of Pilates improved head alignment in the sagittal plane [20]. Additionally, 30–55 min of Pilates therapy has been reported to improve shoulder alignment and muscle mass in middle-aged women [21].

Previous studies have shown significant effects of Pilates on postural alignment, pain, and other physical parameters. However, the duration of the Pilates intervention varied in each study, and it was necessary to investigate the effect of time on the intervention. Furthermore, there have been no studies on the effectiveness of Pilates interventions for UCS, a common condition caused by poor posture in the modern population. Moreover, with little time available for exercise, it is important to identify effective and efficient exercise times to prevent musculoskeletal disorders. Therefore, this study aimed to determine the effects of Pilates exercise on static posture, strength, and muscular endurance in patients with UCS.

## 2. Materials and Methods

### 2.1. Participants

This study was conducted at the G University in Inchon, Republic of Korea, and the study period was between July and October 2021 (12 weeks). All participants were informed of the purpose and methods of the study, and they voluntarily signed an informed consent form. Inclusion criteria for this study were 20–40 years, no participation in Pilates or other exercise within six months, and a craniovertebral angle (CVA) of ≤50° and a kyphosis angle (KA) of ≥24° in the thoracic spine, as determined by UCS. Exclusion criteria included abnormal heart rate and blood pressure; neurological pathologies; rheumatism; pregnancy; regular use of medications, steroids, or muscle relaxants for pain relief within the last six months; and spine-related diseases (diagnosed or surgery). Using the G*Power 3.1 version program (Franz Faul, University Kiel, Germany), an effect size (ES) of f = 0.50, α = 0.05, β = 0.20, and 80% power, a sample size of 21 was calculated. To account for possible missing data and a 30% loss from participants missing follow-ups, we recruited a total of 28 participants. However, 2 participants who missed more than 3 sessions of the Pilates exercise program or more than 1 measurement were excluded from the analysis. Therefore, a final 26 participants were included in the study.

This study was reviewed and approved by the Institutional Review Board of Gachon University (IRB number: 1044396-202106-HR-134-01). We registered with the Clinical Research Information Service in compliance with the World Health Organization regulations (KCT0009274).

### 2.2. Outcomes

Outcome variables were cervicothoracic sagittal alignment, muscle strength, and endurance. Each outcome variable was measured pre-, mid- (4 and 8 weeks), and post-treatment (12 weeks). All measurements were performed in a blinded manner. Two master’s degree measurers who had used Humac NORM for >2 years evaluated cervicothoracic sagittal alignment, muscle strength, and endurance.

#### 2.2.1. Cervicothoracic Alignment

##### Photographic Method Analysis

**Figure 1:**
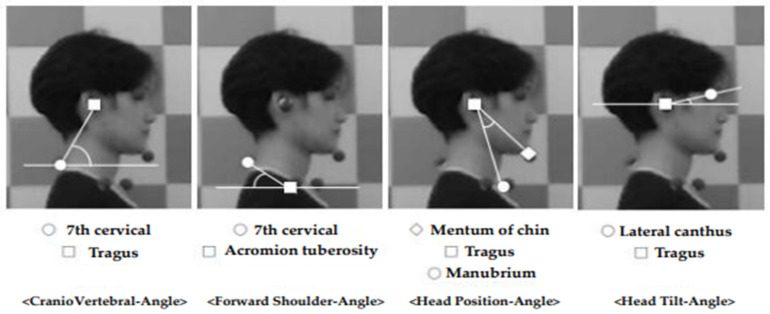
Photographic method analysis of CVA, FSA, HPA, and HTA. CVA, craniovertebral angle; FSA, forward shoulder angle; HPA, head position angle; HTA, head tilt angle.

Figure 1 shows a photographic analysis using a BodySytle analyzer (MZEN, Seoul, Republic of Korea) to measure the CVA, forward shoulder angle (FSA), head position angle (HPA), and head tilt angle (HTA) [22]. The intraclass correlation coefficient (ICC) for photographic methods, such as the BodyStyle analyzer, is 0.99 [23].

The CVA was photographed with markers at the 7th cervical spine and tragus. The software (BodyStyle S8.0: MZEN, Seoul, Republic of Korea) was then used to draw a line parallel to the floor through the 7th cervical spinous process and a straight line between the 7th cervical spine and tragus, recording the angle at the point of intersection [24]. In general, when the CVA is ≤50°, there is an expected misalignment of the lower cervical spine in the UCS outside the normal range [25].

A forward shoulder angle was performed after marking the acromial tuberosity and the 7th cervical spinous process. A line parallel to the floor passing through the acromion tuberosity, a straight line between the acromial tuberosity and the 7th cervical vertebra, and the angle at the point of intersection were recorded [26]. In general, the thoracic spine is considered to have an increased kyphotic angle in the UCS when the FSA is >52°, which is outside the normal range [27].

The HPA was photographed after the markers were placed at the mentum of the chin, tragus, and manubrium. A straight line was drawn through the tragus and manubrium, and the angle was formed by the intersection of the straight line through the tragus and mentum of the chin [28]. In general, an HPA of ≥19.5° is considered outside the normal range and indicative of a cervical spine problem in UCS [28].

Head tilt angle was measured after the lateral canthus and tragus were marked. A straight line was drawn parallel to the floor through the tragus, and a straight line connecting the tragus and lateral canthus was drawn to record the angle at the point of intersection [29]. In general, an HTA of ≤17.20° is outside the normal range, and it indicates a misalignment of the upper cervical spine in the UCS [30]. HTA is correlated with CVA. As CVA decreases, HTA increases as a result of a compensatory pattern of increased flexion of the lower cervical spine and simultaneous extension of the upper cervical spine due to the need to look forward [31].

##### Thoracic Kyphosis Analysis

**Figure 2:**
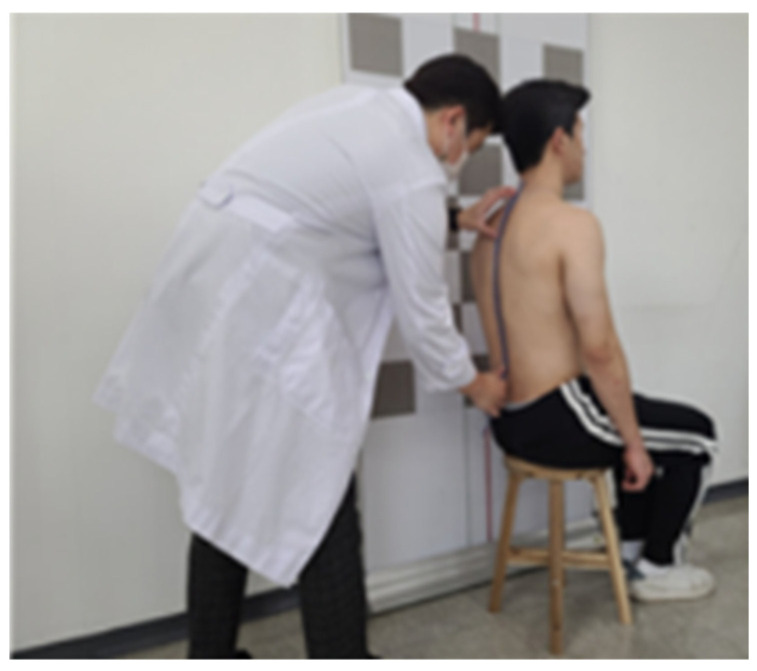
Flexicurve ruler analysis method for thoracic kyphosis angle.

A flexicurve ruler (Staedtler Mars Inc., Nurnberg, Germany) was used to measure the thoracic kyphosis angle. The spinous processes of the 7th cervical and 12th thoracic vertebrae were marked with a marker, and a flexicurve ruler was placed against the spinous processes to obtain a curved shape of the spine (Figure 2). On the curve drawn on graph paper, the points corresponding to the 7th cervical and 12th thoracic vertebrae were marked; the two points were connected with a straight line to record the thoracic length (TL); and the TL was connected vertically from the most convex point backward from the thoracic vertebra to record the thoracic width (TW) [32]. The formula for calculating kyphosis is 4 arctan [(2 × TW)/TL]; if the kyphotic angle is >24°, the thoracic spine is considered hyperkyphotic [33,34]. The resulting values were measured three times and averaged to reduce errors. The flexicurve ruler ICC for thoracic kyphosis is 0.87–0.94 [35].

#### 2.2.2. Muscle Strength and Endurance

**Figure 3:**
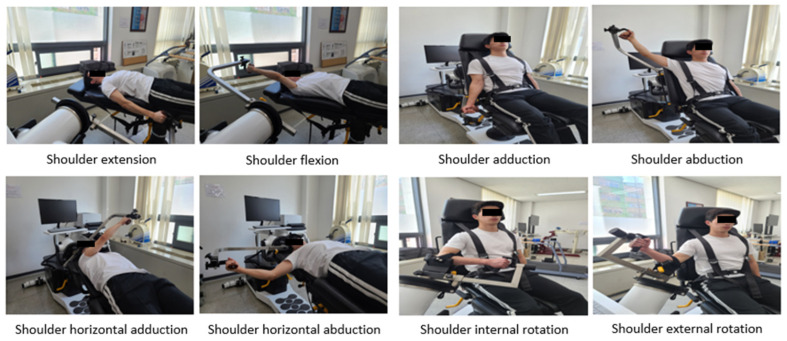
Measurement of muscle strength and endurance of shoulder joint.

Muscle strength and endurance were measured using Humac NORM (CSMi, Stoughton, MA, USA), which quantifies muscle strength and endurance by setting the angular velocity and repetitions [36]. Shoulder flexion and extension, shoulder adduction and abduction, shoulder horizontal adduction and horizontal abduction, and shoulder internal and external rotations were measured, as shown in Figure 3. Prior to the measurement, all the participants stretched in a motion similar to the measurement position to prevent injury and warmed up on a seated cycle at low intensity [37]. Two angular velocities (60 and 180°/s) were used for each shoulder movement. Muscular strength and endurance were measured using the participants’ dominant arm. Strength was recorded by measuring five repetitions at 60°/s (previously practiced three repetitions), and endurance was recorded by measuring 15 repetitions at 180°/s (previously practiced five repetitions) [38]. The rest period between the strength and endurance measurements was at least 1 min. Additionally, the rest period between different movement measurements was at least 5 min to ensure that muscle fatigue did not affect the results [36,39]. The ICC for shoulder muscle strength and endurance test with Humac NORM is 0.72–0.94 [36].

### 2.3. Intervention

The basis of the movements in the Pilates program in this study is Gray Cook’s joint-by-joint concept, which divides the stability and mobility requirements of each joint into parts and then divides them into relaxation and strengthening strategies for each muscle, as shown in Table 1.

The progression of the Pilates exercise program used in this study is divided into phases 1–3, each of which has an objective. Phase 1 (weeks 1–4) aimed to induce relaxation in the upper limbs and trunk so that the subject could perform the exercise program efficiently during the total intervention period. This consisted of self-stretching using a form-roller and massage ball to relax tension in the shoulders, scapulae, upper cervical spine, lower cervical spine, and thoracic spine. Phase 2 (weeks 5–8) focused on increasing the activation of the core musculature and improving muscle activation and movement of the scapula, upper cervical, and lower cervical segmental muscles to improve their stability. Phase 3 (weeks 9–12) consists of strength-oriented exercises to increase the shoulder, cervical, and thoracic muscles to improve mobility in these areas. Subjects performed the program during each study period, with exercise intensity reduced in the event of personal conditioning issues or temporary musculoskeletal problems. All exercise programs were performed at a non-painful exercise intensity, and the researchers moderating the exercise programs provided ongoing reminders of exercise intensity and precautions to subjects throughout the program.

The instructors were certified in Pilates and exercise instruction and had at least three years of experience. Two of the three groups followed the same Pilates program (Table 1) for 12 weeks. The two Pilates groups performed 60 and 30 min of exercise per session. A 60 min Pilates group (60 PG) had a 60 min workout with 10 repetitions and 4 sets, while a 30 min Pilates group (30 PG) had a 30 min workout with 10 repetitions and 2 sets of the same program as the 60 PG. A control group (CG) received one training session with an instructor on how to perform the exercises properly. The CG performed the Pilates program for 12 weeks.

### 2.4. Data Analysis

**Figure 4:**
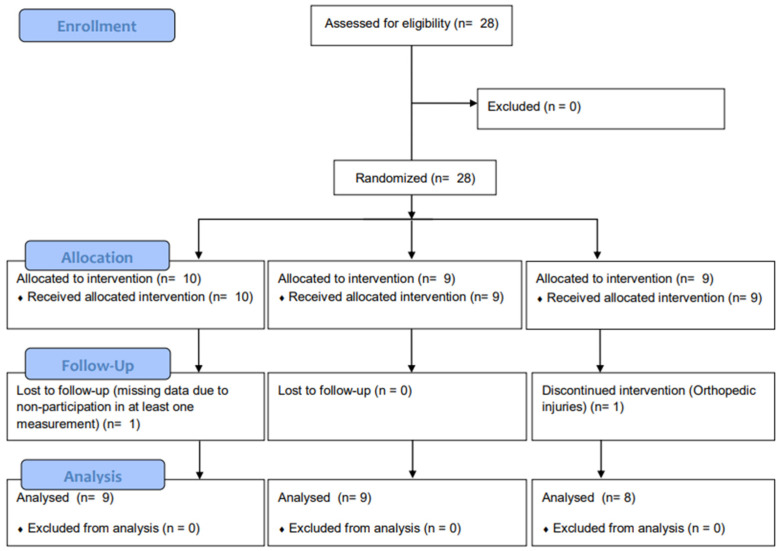
CONSORT flow diagram.

Data analyses were performed using SPSS software (version 26.0; IBM SPSS Inc., Armonk, NY, USA). First, the normality test was performed using the Shapiro–Wilk test. Means and standard deviations were also calculated, and a two-way analysis of variance was performed to determine changes over time and between groups. The Bonferroni test was used to confirm post hoc analysis, and the Hedges’ g calculation formula was used to determine the ES of the Pilates exercises between the groups. Muscle strength and endurance measured with Humac NORM were converted to Newton meters by multiplying the raw data by 1.356, with foot-pounds as the unit of output [40]. The significance level for all statistical analyses was established at 0.05 (*p* < 0.05). This manuscript adheres to the applicable CONSORT guidelines (Figure 4).

## 3. Results

### 3.1. General Characteristics of Participants

Participants were divided into three groups: 60 PG, 30 PG, and CG. Table 2 presents the participants’ general characteristics. There were no significant differences in any variable between the groups (*p* > 0.05).

### 3.2. Cervicothoracic Sagittal Alignment

Table 3 shows the within- and between-group changes in the cervicothoracic sagittal alignment after 12 weeks of participation in the Pilates program. The within-group difference in CVA was that the angle increased over time for both the 60 and 30 PG, with no difference in the CG. The difference between the groups was that 60 and 30 PG increased angulation more than the CG after four weeks. The forward shoulder angle showed no statistically significant differences. The within-group difference in HPA was that the 60 PG showed a decreased angle starting at 12 weeks, while the 30 PG showed a decreased angle between 8 and 12 weeks. There was no difference between the groups until four weeks; at eight weeks, the 60 PG reduced the angle more than the CG, and at 12 weeks, the 60 and 30 PG reduced the angle more than the CG. The difference within the HTA group was that the angle increased at four and eight weeks for the 60 PG but remained the same at 8 and 12 weeks. The 30 PG showed no difference at four weeks and increased angulation at 8 and 12 weeks. The difference between the groups was not different at four weeks, but at eight weeks, the 60 PG increased angulation more than the CG, and at 12 weeks, both the 60 and 30 PG increased angulation more than the CG. The kyphosis angle showed that the within-group differences were that the 60 PG showed a decrease in angle over time, while the 30 PG showed a significant decrease in angle at eight weeks. Between-group differences showed that the 30 PG reduced angulation more than the CG at four weeks, and the 60 and 30 PG angulation were less than the CG after 8 and 12 weeks.

### 3.3. Muscle Strength and Endurance

The results of muscular strength and endurance in the Humac NORM were based on the participant’s dominant arm.

#### 3.3.1. Muscle Strength

Table 4 shows the within- and between-group changes in muscle strength after 12 weeks of participation in the Pilates program. Shoulder flexor strength increased at 8 and 12 weeks compared to that at four weeks in the 60 PG. In the 30 PG, shoulder flexor strength increased at 8 and 12 weeks from baseline, and at four weeks, CG did not change, and there was no difference between the groups. Shoulder horizontal abductor strength increased at 8 and 12 weeks from baseline and at four weeks in the 60 PG. In the 30 PG, shoulder horizontal abductor strength increased at 8 and 12 weeks after four weeks, and the CG did not change; however, it was higher in the 60 PG than in the CG at eight weeks. The strengths of the shoulder extensors, abductors, adductors, horizontal adductors, internal rotators, and external rotators showed no statistically significant changes.

#### 3.3.2. Muscle Endurance

Table 5 shows the within- and between-group changes in muscle endurance after 12 weeks of participation in the Pilates program. Within the muscle endurance groups for shoulder extensors, the 30 PG showed the biggest change at 12 weeks compared with the other time points. The CG showed a decrease in muscle endurance from baseline, while the 60 PG showed no change. In terms of within-group changes in shoulder flexor muscle endurance, the 60 PG was higher at 12 weeks than at 8 weeks. However, the between-group difference was higher in the 60 PG compared to that in the CG at 8 and 12 weeks. For within-group changes in shoulder horizontal abductor muscle endurance, the 60 PG was higher at 8 and 12 weeks than at baseline and 4 weeks, and the 30 PG was higher at 8 and 12 weeks than at 4 weeks. However, the CG was lower at 8 and 12 weeks than at baseline. The between-group difference in shoulder flexor muscle endurance was higher in the 60 PG than in the CG at 12 weeks.

## 4. Discussion

This study aimed to determine the effects of the Pilates program on cervicothoracic sagittal alignment, muscle strength, and endurance in university students with UCS. The effect of the Pilates program and duration of the workout were determined by dividing the time per session into 60 and 30 min when participating in the Pilates program at a frequency of twice a week for 12 weeks. The results showed that the Pilate program improved cervicothoracic sagittal alignment due to UCS and increased strength and muscular endurance in some shoulder movements.

The most prominent problematic posture in UCS is the forward head posture. Forward head posture can be identified using CVA. In this study, CVA = ES: 3.18 (60 PG) vs. ES: 8.27 (30 PG), with significant improvement in both groups. Similar to our results, previous studies have shown improvement in CVA (ES: 3.60) in adult men with UCS using the National Academy of Sports Medicine (NASM)-designed exercise program for 50 min per session, three times per week for eight weeks [41], and improvement in CVA (ES: 1.25) in middle-aged women for 60 min per session, three times per week for eight weeks [25]. In contrast, a program with the specific purpose of scapular stabilization exercises showed a positive effect (ES: 0.67) on CVA in middle-aged women with UCS, even if the exercises were performed for as little as 20 min per session for four weeks. The higher ES in this study than in previous studies may be due to the fact that the exercises included cervical spine-specific movements as well as scapular relaxation, stability, mobility, and strengthening exercises.

The forward head posture that occurs in UCS can also lead to hyperkyphosis of the thoracic spine. Thoracic hyperkyphosis was identified using the KA. The results showed KA = ES: 3.30 (60 PG) vs. ES: 3.04 (30 PG) for both groups, which was a significant improvement. These results were similar to the ESs of yoga (ES: 3.27), the NASM exercise program (ES: 3.37), and the corrective exercise program (ES: 5.18) in adult men and women with UCS for eight weeks at a frequency of 50–60 min per session, three times per week [6,25,40]. Other studies have reported improvement in KA (ES: 0.61) with scapular stabilization exercises three times a week for four weeks, with an exercise duration of <30 min [42], and in women with UCS after coronary artery bypass graft surgery, it was stretching exercises with a gradual increase in duration from 30 to 55 min over eight weeks three times a week with a small ES (ES: 0.07) [43].

Forward head posture and thoracic hyperkyphosis that occur in UCS can be improved with stretching, corrective exercises, yoga, and stabilization exercises. However, the level of ES in the stretching study was small [43]. Furthermore, it is unclear whether stretching alone improves postural alignment or muscle function in existing studies [10], so caution should be used in interpreting the results. In this study, a Pilates program of 30 min per session, twice a week, was found to be effective. This suggests that the Pilates program is as effective as other exercise programs in improving cervical and thoracic postures at UCS. However, in this study, the ES of KA improvement was similar between 30 and 60 min of the Pilates exercise over 12 weeks. Additionally, rather than applying simple exercises, such as scapular stabilization and stretching, a complex exercise program that considers the body’s different conditions seems to be more effective in improving KA.

In this study, FSA, HPA, and HTA were also analyzed to identify the cervicothoracic sagittal alignment in UCS. The results showed significant improvements in the HPA and HTA but no difference in the FSA. Head tilt angle and HPA are commonly associated with forward head posture in the UCS [28]. In particular, the HTA is an angular measure of the degree to which the UCS compensates by hyperextending the head and cervical spine to look forward when the upper cervical spine is flexed [44,45]. Ultimately, the improvements in CVA and KA indicated an improved forward head posture of the UCS. Additionally, changes in postural alignment may reduce the cervical and thoracic compensations, resulting in improved HPA and HTA.

The results and discussion of the effects of muscle strength and endurance after a 12-week Pilates program are as follows: Muscle strength showed an ES for the shoulder flexors (ES: 0.13 [60 PG] vs. ES: 0.65 [30 PG]) and shoulder horizontal abductors (ES: 0.57 [60 PG] vs. ES: 0.41 [30 PG]). Muscle endurance showed ESs for the shoulder extensors (ES: 0.07 [60 PG]), shoulder flexors (ES: 0.07 [60 PG] vs. ES: 0.32 [30 PG]), and shoulder horizontal abductors (ES: 0.56 [60 PG] vs. ES: 0.32 [30 PG]). In another study of an exercise program for UCS, middle-aged women with UCS who performed scapular stabilization exercises three times a week for 15–20 min for four weeks showed increased manual muscle test values for the rhomboid (ES: 3.62), middle trapezius (ES: 2.01), lower trapezius (ES: 2.62), and serratus anterior (ES: 2.58) muscles [42]. A comprehensive and selective corrective exercise program of 50 or 60 min three times per week for eight weeks in adult UCS men showed increases in percentage maximum voluntary isometric contraction of the middle trapezius (ES: 0.06–1.89), lower trapezius (ES: 0.91–1.91), and serratus anterior (ES: 1.52–2.03) muscles [6,46]. The increased strength and muscular endurance of the shoulder flexors in the Pilates program in this study may be a result of the increased strength and muscle activity of the middle and lower trapezius and serratus anterior muscles found in previous studies, as appropriate exercises for UCS require scapular upward rotation, which occurs when the arm is raised.

This study also found increased strength and endurance of the shoulder horizontal abductors. This had a positive effect on the shoulder horizontal abductor, as changes in the posture and structure of the sagittal plane resulted in increased strength and endurance of the shoulder flexor and influenced other planes [47]. However, some strength and muscular endurance factors tended to remain the same or decrease from baseline to 4 weeks and then improve from 8 to 12 weeks. Previous studies have reported unclear strength and muscular endurance gains from stretching alone [10]. Therefore, the results of this study may be due to the fact that the Pilates program was designed and performed with stretching-oriented movements from 1 week to 4 weeks (phase 1).

Based on previous studies and this study, the Pilates program emphasizes breathing and postural alignment, which, if followed correctly, can improve posture [48]. Additionally, a properly designed Pilates program will not be as effective as other exercise programs in improving fitness factors such as strength, endurance, balance, and flexibility [49]. Therefore, the design and application of the Pilates program with appropriate movements for UCS can have a positive impact on posture, strength, and endurance. Furthermore, although a 60 min Pilates program at a frequency of twice a week had a positive effect on UCS, we found that even 30 min of Pilates exercise was sufficient to achieve the same benefits.

However, this study has some limitations. First, the small sample size made it difficult to generalize the results of this study. Second, the 12-week Pilates program used in this study included a combination of movements with no conceptual distinction, making it difficult to determine the effect of each movement on the outcomes. Third, the participants in this study were university students in their 20s; therefore, it is unlikely that the same effects would be seen in older adults and individuals with certain disease conditions (muscle weakness, neurological disorders, or musculoskeletal disorders). Therefore, it is necessary to apply a specific exercise program to UCS of different age groups and disease conditions to confirm its effectiveness.

## 5. Conclusions

This study is important for determining the effect of the Pilates program on cervicothoracic sagittal alignment, muscle strength, and endurance in UCS. The same program was randomized into 60 PG, 30 PG, and CG. Based on the results between groups, the following conclusions were drawn:

First, the cervicothoracic sagittal alignment of the UCS after 12 weeks of participation in the Pilates program showed statistically significant improvements in CVA, HPA, HTA, and KA in the 60 and 30 PG, but not FSA. There was no difference in the cervicothoracic sagittal alignment angle between the 60 and 30 PG.

Second, the muscle strength of the shoulder flexors and horizontal abductors showed statistically significant improvements after 12 weeks of the Pilates program in the UCS group. Muscle endurance showed statistically significant improvements in the shoulder extensors, flexors, and horizontal abductors.

When comparing the ES of the changes in cervicothoracic sagittal alignment, muscle strength, and endurance between the exercise program in this study and previous studies, the Pilates program in this study was found to be as effective as the other exercise programs for UCS. Furthermore, it has been shown that 30 min of exercise twice a week can provide as many benefits as 60 min of exercise. Therefore, the Pilates program designed for the characteristics of UCS is needed to encourage individuals to participate in even 30 min of exercise.

## Figures and Tables

**Table 1:** Pilates program.

Phase	Object	Segment	Target Muscle and Movement	Exercise Name	Repetition (Time)/Set
1 phase (1–4 weeks)	Release	Shoulder	Pectoralis major Latissimus dorsi	Shoulder warm-up (F)	60 PG = 30 s/4 set 30 PG = 30 s/2 set
				Scapula mobility (F)	
				Lat massage (F, M)	
				Kneeling cat (F)	
		Scapula	Rhomboid	Upper back massage (F, M) Chest massage (M)	
			Levator scapula		
			Pectoralis minor		
		Upper cervical	Suboccipital	Neck massage (F, M)	
			Longissimus capitis		
			Splenius capitis		
		Lower cervical	Sternocleidomastoid	Self-stretching	
			Scalenes		
		Thoracic	Rotation	Spine torso rotation	60 PG = 10 reps/4 sets
				Seated side stretch	30 PG = 10 reps/2 sets
2 phases (5–8 weeks)	Stability	Scapula	Trapezius Serratus anterior	Swimming Spine twist (B) Salute (B) Punching (B)	60 PG = 10 reps/4 sets 30 PG = 10 reps/2 sets
		Upper cervical	Longus capitis Longus colli Recuts capitis Suprahyoid	Single leg kick Double leg kick	
		Lower Cervical	Splenius cervicis Longissimus cervicis Semispinalis cervicis	Chicken wing	
3 phases (9–12 weeks)	Mobility and Reinforce	Shoulder	Deltoid	Spine twist (B) Arm work series (B)	60 PG = 10 reps/4 sets 30 PG = 10 reps/2 sets
		Cervical	Extension	Swan	
		Thoracic	Flexion	Upper back curl up (F) Rolling like a ball	
			Lateral flexion	Mermaid Saw	

Notes: F, form roller; M, massage ball; B, resistance band; 60 PG, 60 min Pilates group; 30 PG, 30 min Pilates group.

**Table 2:** General characteristics of participants.

Factor	60 PG (*n* = 9)	30 PG (*n* = 9)	CG (*n* = 8)	*p*-Value
Sex (male/female)	5/4	2/7	2/6	0.286
Age (years)	22.78 ± 1.79	22.33 ± 1.41	23.00 ± 2.67	0.782
Height (cm)	168.56 ± 9.48	165.22 ± 5.65	165.75 ± 10.12	0.682
Weight (kg)	61.22 ± 11.42	60.78 ± 11.32	62.38 ± 14.34	0.964
BMI (kg/m^2^)	21.39 ± 2.67	22.15 ± 3.12	22.46 ± 2.74	0.728
CVA (°)	47.09 ± 3.28	48.83 ± 0.98	46.15 ± 3.80	0.174
FSA (°)	41.10 ± 6.41	39.13 ± 6.15	40.02 ± 8.51	0.839
HPA (°)	31.67 ± 2.63	33.15 ± 5.30	32.82 ± 3.08	0.595
HTA (°)	12.21 ± 3.12	12.51 ± 1.64	13.40 ± 1.95	0.564
KA (°)	54.14 ± 12.19	44.70 ± 7.94	45.75 ± 9.32	0.115

Note: 60 PG, 60 min Pilates group; 30 PG, 30 min Pilates group; CG, control group; BMI, body mass index; CVA, craniovertebral angle; FSA, forward shoulder angle; HPA, head position angle; HTA, head tilt angle; KA, kyphosis angle.

**Table 3:** Differences in cervicothoracic sagittal alignment between Pilates program groups.

Factor	Group	Baseline	4 Weeks	8 Weeks	12 Weeks	F (*p*)	Effect Size (Hedges’ *g*) Baseline to 12 Weeks
CVA (°)	60 PG	47.09 ± 3.28	50.01 ± 2.43	55.88 ± 4.58	61.24 ± 5.36	30.539 *** (<0.001)	−3.18
	30 PG	48.83 ± 0.98	51.12 ± 2.77	54.70 ± 2.56	63.34 ± 2.28		−8.27
	CG	46.15 ± 3.80	46.68 ± 3.11	49.81 ± 2.78	46.08 ± 3.32		0.02
FSA (°)	60 PG	41.10 ± 6.41	44.21 ± 5.18	43.98 ± 5.36	43.35 ± 4.37	1.030 (0.413)	−0.41
	30 PG	39.13 ± 6.15	41.89 ± 6.59	42.00 ± 6.72	42.39 ± 3.45		−0.65
	CG	40.02 ± 8.51	40.80 ± 7.04	37.99 ± 4.61	37.81 ± 5.51		0.31
HPA (°)	60 PG	31.37 ± 2.63	32.01 ± 3.30	30.10 ± 3.88	26.37 ± 3.51	3.225 ** (0.008)	1.61
	30 PG	33.15 ± 5.30	34.73 ± 3.59	31.83 ± 3.53	27.16 ± 2.40		1.46
	CG	32.82 ± 3.08	33.73 ± 3.49	35.15 ± 4.91	33.89 ± 4.45		−0.27
HTA (°)	60 PG	12.21 ± 3.12	13.70 ± 2.71	16.69 ± 2.15	18.16 ± 1.59	5.657 *** (<0.001)	−2.40
	30 PG	12.51 ± 1.64	12.70 ± 2.16	15.29 ± 1.65	18.27 ± 2.54		−2.69
	CG	13.40 ± 1.95	12.76 ± 1.99	13.33 ± 3.17	12.82 ± 4.61		0.16
KA (°)	60 PG	54.14 ± 12.19	43.38 ± 7.12	30.69 ± 11.08	22.38 ± 6.01	12.480 *** (<0.001)	3.30
	30 PG	44.70 ± 7.94	40.84 ± 6.87	27.52 ± 4.38	25.21 ± 4.38		3.04
	CG	45.75 ± 9.32	48.75 ± 6.26	50.63 ± 8.12	49.94 ± 3.73		−0.59

Note: CVA, craniovertebral angle; FSA, forward shoulder angle; HPA, head position angle; HTA, head tilt angle; KA, kyphosis angle; 60 PG, 60 min Pilates group; 30 PG, 30 min Pilates group; CG, control group; ** *p* < 0.01; *** *p* < 0.001.

**Table 4:** Differences in muscle strength between Pilates program groups.

Factor	Group	Baseline	4 Weeks	8 Weeks	12 Weeks	F (*p*)	Effect Size (Hedges’ *g*) Baseline to 12 Weeks
Extensor (N)	60 PG	48.39 ± 24.20	44.53 ± 21.78	45.57 ± 21.78	44.68 ± 19.13	0.609 (0.722)	0.17
	30 PG	34.39 ± 16.18	29.54 ± 11.06	36.22 ± 13.78	33.10 ± 10.83		0.09
	CG	35.73 ± 17.47	36.23 ± 22.00	37.90 ± 21.94	36.73 ± 31.15		−0.04
Flexor (N)	60 PG	36.22 ± 17.64	30.43 ± 12.22	36.51 ± 13.19	38.44 ± 15.82	3.561 ** (0.004)	−0.13
	30 PG	25.68 ± 10.01	25.232 ± 7.30	31.47 ± 8.81	31.61 ± 8.29		−0.65
	CG	31.39 ± 12.41	31.73 ± 14.94	30.56 ± 12.31	28.39 ± 10.92		0.26
Abductor (N)	60 PG	29.09 ± 11.93	29.68 ± 8.15	34.58 ± 10.70	35.62 ± 11.76	2.037 (0.072)	−0.55
	30 PG	28.83 ± 9.42	24.94 ± 5.43	29.98 ± 10.56	31.76 ± 7.98		−0.34
	CG	27.72 ± 10.85	26.38 ± 9.49	28.89 ± 10.44	26.22 ± 6.14		0.17
Adductor (N)	60 PG	36.22 ± 24.48	34.73 ± 19.78	36.07 ± 17.72	36.36 ± 14.15	1.465 (0.203)	−0.01
	30 PG	25.53 ± 21.91	22.56 ± 9.71	31.17 ± 11.06	32.21 ± 7.51		−0.41
	CG	23.71 ± 28.25	27.38 ± 17.62	23.88 ± 15.83	19.70 ± 14.52		0.18
Horizontal abductor (N)	60 PG	30.13 ± 15.31	29.09 ± 10.68	36.07 ± 13.87	39.63 ± 17.71	4.176 ** (0.001)	−0.57
	30 PG	29.24 ± 14.88	23.01 ± 2.29	31.91 ± 11.21	33.50 ± 13.88		−0.30
	CG	28.72 ± 15.64	26.38 ± 12.30	23.54 ± 10.94	23.21 ± 17.17		0.36
Horizontal adductor (N)	60 PG	37.85 ± 23.72	35.92 ± 15.16	40.07 ± 18.29	39.33 ± 18.49	1.373 (0.238)	−0.07
	30 PG	26.42 ± 7.87	23.30 ± 3.48	27.61 ± 10.95	31.47 ± 11.47		−0.51
	CG	27.72 ± 12.05	28.55 ± 12.14	26.05 ± 11.80	25.38 ± 19.24		0.16
Internal rotator (N)	60 PG	32.06 ± 16.35	32.21 ± 14.06	32.50 ± 13.22	32.65 ± 10.98	0.432 (0.855)	−0.04
	30 PG	28.20 ± 9.41	26.27 ± 6.75	28.65 ± 10.04	27.90 ± 6.17		−0.04
	CG	27.38 ± 16.25	26.38 ± 9.55	25.71 ± 8.94	25.21 ± 8.06		0.17
External rotator (N)	60 PG	7.86 ± 5.68	8.76 ± 5.05	10.69 ± 4.95	13.36 ± 6.58	1.526 (0.183)	−0.89
	30 PG	5.64 ± 4.71	7.12 ± 4.22	9.49 ± 6.35	10.54 ± 4.88		−1.02
	CG	8.84 ± 6.58	7.51 ± 7.19	7.68 ± 6.01	8.68 ± 6.54		0.03

Note: N, newton-meter; 60 PG, 60 min Pilates group; 30 PG, 30 min Pilates group; CG, control group; ** *p* < 0.01.

**Table 5:** Differences in muscle endurance between Pilates program groups.

Factor	Group	Baseline	4 Weeks	8 Weeks	12 Weeks	F (*p*)	Effect Size (Hedges’ *g*) Baseline to 12 Weeks
Extensor (N)	60 PG	36.66 ± 18.02	33.54 ± 16.48	35.18 ± 16.14	36.66 ± 17.33	3.907 ** (0.002)	0.00
	30 PG	22.56 ± 12.66	24.64 ± 9.50	29.68 ± 12.04	27.46 ± 10.09		−0.43
	CG	26.88 ± 15.40	25.71 ± 16.79	24.71 ± 16.38	22.71 ± 17.36		0.25
Flexor (N)	60 PG	32.36 ± 13.07	27.76 ± 9.35	30.43 ± 9.60	31.47 ± 11.20	3.075 * (0.010)	0.07
	30 PG	22.56 ± 6.49	24.79 ± 6.02	28.50 ± 7.47	29.39 ± 9.75		−0.82
	CG	28.39 ± 7.20	29.72 ± 11.53	29.89 ± 8.26	26.55 ± 10.70		0.20
Abductor (N)	60 PG	31.47 ± 14.09	36.22 ± 9.34	36.81 ± 6.88	35.62 ± 9.61	0.689 (0.659)	−0.34
	30 PG	33.10 ± 11.81	31.76 ± 7.67	37.25 ± 6.35	37.25 ± 7.97		−0.41
	CG	30.06 ± 9.12	27.22 ± 10.49	35.57 ± 11.31	31.73 ± 7.72		−0.20
Adductor (N)	60 PG	29.68 ± 21.76	29.68 ± 14.29	30.43 ± 16.67	31.76 ± 16.87	0.858 (0.530)	−0.11
	30 PG	22.86 ± 21.80	14.99 ± 10.32	21.82 ± 15.65	19.15 ± 12.99		0.21
	CG	20.37 ± 18.09	16.70 ± 15.46	13.36 ± 12.55	15.53 ± 15.84		0.28
Horizontal abductor (N)	60 PG	22.71 ± 16.69	23.45 ± 9.11	30.58 ± 11.75	31.76 ± 15.77	4.813 *** (<0.001)	−0.56
	30 PG	24.94 ± 9.23	19.89 ± 4.50	26.27 ± 8.18	28.20 ± 11.02		−0.32
	CG	26.05 ± 12.53	23.54 ± 8.04	21.21 ± 7.47	18.70 ± 9.14		0.67
Horizontal adductor (N)	60 PG	30.43 ± 14.65	30.28 ± 11.41	33.54 ± 13.37	31.47 ± 12.59	1.733 (0.126)	−0.08
	30 PG	23.60 ± 7.07	22.26 ± 4.28	26.57 ± 9.02	29.68 ± 9.60		−0.72
	CG	26.72 ± 9.06	26.88 ± 8.70	24.38 ± 8.53	22.88 ± 12.19		0.36
Internal rotator (N)	60 PG	29.68 ± 17.87	27.61 ± 15.16	28.79 ± 12.64	26.86 ± 12.07	1.634 (0.151)	0.18
	30 PG	19.89 ± 10.49	17.96 ± 7.73	21.82 ± 8.53	38.89 ± 41.00		−0.63
	CG	19.70 ± 14.45	17.70 ± 8.16	20.20 ± 9.98	19.37 ± 9.53		0.03
External rotator (N)	60 PG	8.76 ± 4.68	6.38 ± 4.81	9.05 ± 4.47	11.13 ± 6.84	1.454 (0.207)	−0.40
	30 PG	5.79 ± 4.63	6.53 ± 4.55	5.79 ± 4.22	6.38 ± 3.77		−0.14
	CG	7.37 ± 2.94	7.01 ± 3.25	6.51 ± 3.07	8.68 ± 4.16		−0.36

Note: N, newton-meter; 60 PG, 60 min Pilates group; 30 PG, 30 min Pilates group; CG, control group; * *p* < 0.05; ** *p* < 0.01; *** *p* < 0.001.

## Data Availability

The data presented in this study are available on request from the corresponding author.
